# Feasibility study: one year fortnightly follow-up of the evolution of supra-spinatus degeneration via text-messages

**DOI:** 10.1186/s12998-020-00343-4

**Published:** 2020-11-04

**Authors:** Karl Vincent, Emeritus Olivier Gagey, Charlotte Leboeuf-Yde

**Affiliations:** 1 ED 566, Université Paris-Saclay, 15 r Georges Clemenceau, 91405 Paris, Orsay France; 2 Institut Franco-Européen de Chiropraxie, 24 Bd Paul-Vaillant-Couturier, 94200 Toulouse, Ivry-sur-Seine France; 3 Institute for Regional Health Research, B.Winsløws Vej 19, Dk, 5000 Odense C, Denmark

**Keywords:** Rotator cuff disorders, Supraspinatus, Longitudinal study, Evolution (course), Prognosis, Trajectory, Feasibility study, Text messaging

## Abstract

**Background:**

The clinical follow-up of patients for degeneration of the supraspinatus tendon is limited by the lack of objective assessment of pain evolution over time. We therefore tested a new method to collect follow-up data on patients treated either by surgical cuff repair or rehabilitation.

**Objectives:**

We report the feasibility this method in terms of recruitment of clinicians and patients and their compliance.

**Methods:**

In this multicenter longitudinal observational study, between September 2015 and March 2019, patients consulting either for surgical repair or rehabilitation were examined at baseline and after twelve months by their clinician, including the Mini-DASH questionnaire. Fortnightlys, during one year, patients were asked about number of days their shoulder problem affected their daily life, number of nights woken up from shoulder pain, and present pain score, using text-messages for sending and responding to questions. A system administrator supervised responses and non-compliant subjects were contacted and assisted with the procedure. The CONSORT statement for pilot studies was followed.

**Results:**

Four of 11 invited clinicians accepted participation and collected data till the end. Of the 410 patients we originally planned for, 252 were included in the study, but complete data for the clinicians’ follow-up at 12 months were missing for 30. Of the 222 subjects with SMS data files, 190 (85%) provided at least 80% of their fortnightly messages. All three SMS messages were answered equally often. In total, 160 study subjects answered at least 80% of times *and* had clinical data at twelve months, i.e. 39% of the intended study sample and 72% of the 222 subjects with SMS data.

**Conclusion:**

The most important difficulty of this study was the enrolment and compliance of clinicians. The collection of SMS data was less successful than in previous studies, but French people accepted well this new method which is much easier and specific than collecting data through clinical records. The quality of the SMS data was acceptable. However, because of the limited number of complete datasets, only a limited number of questions from the original study protocol can be answered.

## Background

### The painful shoulder

The rotator cuff consists of a group of four muscles with their tendons that stabilize the shoulder. These are the supraspinatus, infraspinatus, teres minor, and the subscapularis muscles. Painful shoulders associated with degenerative pathologies of the rotator cuff are a public health issue due to their prevalence, cost of care, and the disability they may cause [1, 2]. Epidemiologic studies on the prevalence of musculoskeletal pain indicate that shoulder pain is fairly common, with prevalence estimates as high as 2/3 of the general population over the lifetime [3]. As an example of how costly it is, in 2000, the cost of general shoulder disorders was estimated to be $ seven billion in the United States [4]. Epidemiologic studies also show that degenerative rotator cuff tendons represent the main cause of shoulder pain; as an example, the second cause is traumatic, responsible for only 7% of cases [5]. Once various pathologies of the painful shoulder have been excluded through an anamnesis, clinical examination and possibly other tests such as imaging, the degenerated rotator cuff will often be considered.

### The degenerated rotator cuff

The degeneration of the rotator cuff starts as a subacromial impingement syndrome that is considered as an initial step according to the Neer theory [6]. It evolves into a ‘tendinopathy’, which in 80% of cases affects the supraspinatus tendon. Anatomically, it results in a disorganization of the intimate architecture of the tendon that can be identified by additional examinations such as ultrasound, CT arthrography or MRI. A tendinopathy may progressively get worse by partial or complete tears of one or several tendons [1]. The incidence of rotator cuff tears increases with age with more than half of individuals in their 80s having a rotator cuff tear [3].

### Apparent discrepancy between degeneration and pain

Although a painful shoulder often is believed to be directly linked to one or several degenerated tendons, there is a discrepancy between the degree of degeneration and pain. Thus, we have previously shown that the prevalence of the degenerative rotator cuff lesions increases linearly throughout life and that, although the prevalence of shoulder paint also increases with age, pain is reported to a much lesser extent, to rather diminish in later age [7].

### Treatment of the degenerated shoulder cuff

Treatments of these conditions are mainly conservative (rehabilitation sometimes associated with anti-inflammatory drugs, shoulder injections, manual therapy) or surgical (subacromial decompression with or without cuff repair). Rehabilitation is mainly done first and surgery when conservative treatment fails. But, according to the literature, there is no difference between these two approaches in terms of short or long term outcome [8].

### Assessment of the degenerated rotator cuff

In the case of the degenerated supraspinatus tendon, passive movement of the shoulder is rarely affected, concerning mainly large rotator cuff tears [9]. During the acute phase, however, intensity of pain may cause both diminished active movement and strength [9, 10]. Since these findings are secondary to the pain, the pain in itself is the main parameter in the assessment of the effectiveness of the outcome of treatments.

### The trajectory of pain for the degenerated rotator cuff

Presently, clinical development (the trajectory) of the degenerated rotator cuff is not well understood. Clearly, the clinical study of this condition depends on two major factors: the assessment of pain and its monitoring over the time.

### Monitoring of pain

Therefore, the objective assessment of pain is the biggest challenge of the clinical examination and in research. There are several explanations. In clinical practice, monitoring of pain is quite often « incomplete » because control consultations are necessarily apart. These time intervals may contribute to modify the evaluation of the past pain provided by the patients (memory decay) [11]. Pain is also variable over time and this variability will express itself during the clinical assessment and influence the reporting of the pain considerably [12]. In addition, the interview of the patient conducted by the therapist can influence the patient’s discourse (recall bias) [13]. This synthesis of the pain history is therefore necessarily marked by subjectivity of the patient as well as of the clinician. It is likely that the therapist tends to minimize the pain of the patient following treatment because he wishes his patient to be better and that the patient minimizes his pain in order to satisfy the therapist.

Our knowledge is still unclear regarding the mode of progression of pain, whether in relation to the prognosis after treatment or its natural course. Current conventional surveys or clinical studies have the same problems, as the monitoring of pain is difficult to perform [8]. Typically, baseline data on shoulder pain is viewed as a theoretical starting point regardless the previous duration of the problem. Then, data monitoring and collection are organized for short, medium or long term, at preset intervals (for example every 3 months) that do not make it possible to follow the precise evolution of the pain course [12]. In the same manner, a theoretical time of end of monitoring is decided, but this one may not necessarily match the end of the pathology nor occur at the same time during the evolution of the disease for each participant. When researchers attempt to study more detailed trajectories, patients may be asked to keep a diary. However, this method is not necessarily reliable because patients could forget to include information, and it is therefore probably often done a posteriori [12].

### Collection of data using text messages

Until now, data collection for clinical and research purposes has been through retrospective reporting (last month, last 3 months, last year after the beginning of the treatment) but new technology, using an electronic data collection method, makes it possible to collect frequently longitudinal data cheaply and with a much better compliance [14]. Further, such data can be reported as detailed pain trajectories (described, for example, as: unchanged, rapid improvement, slow improvement, episodic) and, simply, as number of days with pain over the surveyance period.

### Rationale for the study

In conclusion, there are multiple unanswered questions regarding the evaluation and the evolution of pain associated with degeneration of the supraspinatus tendon as well as the choice of the most appropriate treatments and their ranking based on outcome. The use of a better method of collecting data in the clinical setting and in surveys would help providing answers to these questions. We therefore planned a study, in which various treatment approaches would be monitored in a multicenter study, using the answers given on frequent automated text messages (‘SMS’) over 1 year.

This type of data collection has been used in previous studies, particularly in the Nordic countries [12, 14–18]. In our experience, people from the Nordic countries are often willing to follow instructions and to do so over a prolonged time, if given appropriate information. However, this type of data collection has never been carried out in France. Differences between cultures may modify the compliance for different types of data collection both among the clinicians and the patients. Therefore, an initial analysis of the difficulties in recruiting patients and collecting data was of great importance. A previous feasibility analysis performed on chiropractic patients participating in a study on low back pain in Sweden showed that weekly text messages are useful to examine the clinical course of LBP in the primary care sector [19]. Clearly, it would be relevant to perform a feasibility study, to establish if this method would be acceptable to clinicians and patients also in France and in relation to the treatment of shoulder problems.

## Methods/design

### Aim and objectives

The aim of this study was to test the feasibility of this method to collect data for treatments of the degenerated supraspinatus tendon using a dual follow-up: classic medical assessment and SMS track.

The objectives of the study were to report on the various hurdles throughout the data collection that had an effect on the final study sample. Thus, we reported on the number of data collecting clinicians, the number of patients who entered the study and completed the study, separating these by those who i) had complete patient files and ii) those who had complete (> 80%) SMS responses over the year of data collection, and combining these two variables into iii) those who had both. Thereafter, we planned to comment on the problems that were identified, provide remedial suggestions, and, if relevant, give advice on future similar studies undertaken in French populations. The CONSORT statement for pilot studies was followed.

### Study design and setting

This is a multicenter observational study of patients consulting in the primary or secondary sector, treated with current modalities for pain believed to be related to degeneration of the supraspinatus tendon. As this was a multicenter practice-based study, treatment was decided by clinicians according to their usual clinical approach and no attempt was made to guide or monitor their activities. All clinicians filled out a base-line questionnaire provided by the researchers. The clinical monitoring with the treating practitioner was also standardized to take place at baseline and at three, six and 12-months. In addition, a questionnaire was answered by the patient at baseline and the one-year assessment. Meanwhile, patients were surveyed by the research team using SMS every 2 weeks during 1 year from the beginning of the treatment period via three questions with simple quantitative answers.

### Study participants

#### Patients

Patients were recruited consecutively in health practitioners’ offices of the primary or secondary sector. Adult patients, who were treated for any kind of shoulder pain related to degeneration of the supraspinatus tendon, who consulted the participating clinicians, were eligible. Additional inclusion criteria were that they were French speaking, that they had a cell phone, and that they knew how to use text messages.

Non-inclusion criteria were: pain occurring after severe trauma (not including a simple fall on the tip of the shoulder), shoulder instability, presence of neuropathic pain or an associated complex regional pain syndrome, suspicion of frozen shoulder syndrome, presence of glenohumeral osteoarthritis on the radiological report, symptomatic acromio-clavicular osteoarthritis, calcific tendinopathy, diabetes, rheumatic, neurological, inflammatory or secondary pathologies. Patients with severe pathologies, such as cancer under treatment or inflammatory disease were also not included.

Baseline data were recorded by the treating clinicians at the first visit and included personal data, initial examination findings, the mini-DASH questionnaire [20], diagnosis, and (if relevant) reasons for non-inclusion in the study, described in Additional File 1.

#### Clinicians

Clinicians invited to participate consisted of a convenience sample of orthopedic surgeons, other medical practitioners, physiotherapists, and a physiotherapist/chiropractor all with a professional experience exceeding 10 years. Personal contacts were made with these persons, including two meetings for one group of practitioners working in the same center, to create interest and encourage participation. Both the main supervisor (OG) and the research officer (KV) participated in the data collection.

### Recruitment procedure

Patients were recruited by the practitioners who participated in this study. The latter briefly explained the purpose of the research, that baseline and follow-up data would be recorded, and that participants were expected to respond via text messages over a one-year period to simple questions sent on their mobile phone about the pain and daily activities. They were also informed that participation was voluntary and that all data analysis and reporting would be anonymous, making it impossible to identify any participants. Patients who accepted participation were recruited into the study by the treating clinician. Absence of refusal was recorded at the first visit according to French law. To be included in the study, patients had to send a first SMS to the SMS-Track System after having been provided with the telephone number by their clinician. Then, a research assistant would call the participants to explain how to answer the questions. The nature and frequency of the questions were specified. Patients who agreed to participate but failed to transmit their phone number to the SMS-Track System would thus appear to be included in the study but would not have any SMS data, for which reason their data would be unexploitable.

### Classification and diagnosis of the degeneration of the supraspinatus tendon

Diagnosis was based on an orthopedic examination supplemented by basic imaging, usually at least an ultrasound and an X-ray of the shoulder (see Additional File 1). Then, each practitioner classified the patient into one of four groups according to the types of degeneration of the supraspinatus tendon (4):

1. Impingement syndrome: shoulder pain with “normal” X-rays and ultrasound, showing at the most an effusion of the sub-deltoid bursa.
2. Tendinopathy without tear: “normal” X-rays (i.e. no degenerative joint changes) and ultrasound showing tendon lesions (superficial or deep) without any perforation.
3. Incomplete perforating tear of the supraspinatus
4. Complete supraspinatus tear

### Interventions

The patients were treated according to the protocol of care that was normally used by the practitioner who recruited them without any modification (observational current care study). Surgery would either consist of an arthroscopic or open tendon repair procedure, which would typically be performed after a failed rehabilitation treatment or directly in case of a big supraspinatus degenerative tear. The conservative treatment mainly involved rehabilitation, supervised or unsupervised, sometimes accompanied by manual therapy, acupuncture, medication or injections. All previous treatments were noted, as shown in Additional File 1.

### Data collection

#### Data collected by clinicians

##### Initial evaluation

Clinicians filled out an initial assessment form at the first visit, representing the base-line examination, including the patient’s personal data, results of the clinical history, the physical examination, the additional screening tests, and the final diagnosis on the type of lesion encountered (Additional file 1). Regarding complementary tests, we accepted the minimum assessment usually performed, which includes standard antero-posterior and lateral X-Rays and an ultrasound examination of the rotator cuff (Additional file 1). No additional assessment was required. Each practitioner could, however, complete the assessment with additional tests (bone scan or MRI) independently of our data collection.

##### Mini-DASH questionnaire

The initial evaluation included also the mini-DASH questionnaire (Additional file 1). Reliability and validity of this questionnaire has been demonstrated in the context of populations suffering from degenerative rotator cuff disorders [21]. This questionnaire has been translated into French and validated by the official French health authority (“Haute Autorité de Santé”) [20]. Study participants were asked to fill this out at the first visit and at the one-year follow-up visit.

##### Clinical monitoring

An evaluation form was filled out by the practitioner in charge at the three, six and twelve-months follow-up visits (Additional file 1). The personal data at baseline included the study participant’s initials, year of birth, and cell phone number and also sex, age, diabetes, smoking, socio professional level, and type of occupation.

During the monitoring visits, the clinician asked how many days the study participant was bothered by his/her shoulder during the past period and the answers were ranked for analysis into 4 categories: 0 day, 1 to 7 days, 8 to 14 days, 15 to 21 days, or almost every day, according to the “Nordic Musculoskeletal questionnaire” classification [22] (Additional file 1).

#### Additional questions at the twelve months follow-up

At the 12 months visit, study participants were asked to fill out an additional questionnaire, consisting of four questions on the treatment(s) applied, possible sick leave, if the patient continued to perform the painful shoulder movement activities (work or leisure), and shoulder incapacity (Additional file 1). The last question (n°15) on shoulder incapacity was supposed to be similar to the information collected during 1 year by SMS data with two major objectives and would be used to compare the information obtained with these two methods of data collection. Thus, in the case where study participants did not attend their last visit, a research secretary contacted them by phone to propose them to respond orally to the questionnaire. Finally, if we could not reach them by phone, we sent them a letter with the self-report questionnaire to return. This means that we aimed to collect outcome data also from most of the poor responders on SMS Track. Thus, regarding study participants who did not respond (well) to our SMS messages, we were able to compare the evaluations (clinician’s opinion and patient’s fortnightly reporting) to evaluate how they matched.

#### Automated tracking

The SMS Track data collection was used for the duration of the study. The study participants were instructed to answer to the text messages that were sent to them every 2 weeks. These consisted of three questions about ability to perform daily activities, waking up at night due to shoulder pain, and level of pain. The responses via text messages were recorded into an automated database (SMS-Track). This system has shown to have high compliance in the collection of data, especially when prompted answers are short and simple. It has also ensured, in previous research, a very high level of response, up to 90% [12].

It has been noted that it enables an effective collaboration and compliance of the study participants, when clear information is given to them from the start of the study. All data collected by the system was directly and immediately accessible by the administrator who was able to contact participants, who did not answer or gave inappropriate answers.

The questions asked were:

1. How many days did your shoulder bother you from doing your daily life activities during the past 2 weeks? (answer between 0 and 14)
2. How many nights did your shoulder wake you up during the past 2 weeks? (answer between 0 and 14)
3. Score between 0 and 10 the maximum pain you experienced yesterday during the day?

### Organization

Data collection related to the clinical visits were recorded on paper files that were entered in a database after the 12 months assessment. The SMS Track data were automatically entered into a data file without any personal information except the phone number. Non-responders or people who misused the answer instructions were contacted individually by the research assistant and taught how to answer correctly. The only link between the written clinical file to the text-message file was the person’s telephone number. The databases were merged prior to any analysis, and the telephone numbers removed after generation of a random index.

#### Data management

The three text message questions were sent and received from a server as encrypted messages. Collected data were not stored in the server but in a computer without internet connection.

### Variables of potential interest

#### Descriptive variables

Sex, work, sport, and leisure activities, activities with arm-in-the-air, stress during these activities, education level, place of initial consultation when entering the study, type of diagnosis, severity / chronicity of the supraspinatus degenerative lesion, and the mini-DASH disability questionnaire score.

#### Main potential prediction variables

Type of treatment and type of supraspinatus lesion.

#### Outcome variables

Number of days with bothersome pain, number of nights waking up because of pain, number of days with certain (to be defined) level of disability. Different trajectories of pain (to be determined during preliminary analyses of the data). These data will be provided by the SMS-Track software. The levels of disability and pain at the various follow-up times will be determined through the mini-DASH score and additional questions asked by the clinician.

### Statistical analysis

In the main study, the following analyses were planned:

Base-line data: A descriptive analysis will be done of the clinical and demographic data on the participants. The follow-up data involving the Mini-DASH questionnaire will be reported at the end of the follow-up visits and compared to the base-line scores.

The SMS data will be used to determine the evolution of the supraspinatus painful lesion, to establish subgroups that can be treated as discrete variables. Such subgroups could be described based on their trajectory curves over 1 year, describing for example, time to improvement and time to new events, They can also be used to classify different patterns in relation to pain reporting, such a presence of more or less constant pain, presence of intermittent pain, almost no pain. The total number of days of pain can be taken into account as well as the individual evolution curves over the one-year period. The analyses will therefore depend to a large degree on the look of the data.

#### Determination of sample size

In the perspective of a multivariate analysis, the basic sample is estimated at 50 patients. Each possible response to the additional different variables studied increases the size of ten subjects (i.e. sex: man *n* = 10, woman *n* = 10) [23]. The number of analyses will therefore depend on the final sample, which we hoped would reach 410 to allow for 18 additional variables if they all have two answering categories (Additional file 2).

#### Comparison of the evolution of pain using the two methods

The total number of days with pain recorded with text messages will be compared to the pain duration at the 12 months follow-up visit as estimated by the clinicians, using percentages with a 95% confidence interval. The type of pain evolution over time reported with text-messages will be assessed by visual analysis and compared to the final category reported by the physician (better, stationary etc.). Independent prognostic parameters (diagnosis, treatment group) will be tested in a bivariate analysis. Ideally, other variables (age, sex, socio-professional level, mini-DASH score) should be tested in a multivariate analysis to search for independent relationships with the two treatments groups and the major diagnostic groups, also controlling for treating clinician.

### Ethical considerations and information to participants

This observational study did not present any specific risk since patient care did not differ from the current practice of each practitioner involved in the study. All received text messages were encrypted and the data were totally anonymized before any analysis by substitution of the phone number by a random index number, therefore, the results were analyzed and reported anonymously. In the paper files completed by the practitioner the phone numbers as well as the initial letters of the name and surname of the patients were removed physically, this means that also paper files are rigorously anonymous.

The three neutral encrypted questions sent by SMS and the answers, leading to three meaningless numbers that were sent back at random time, were very unlikely to be intercepted and used maliciously by external persons.

Given the use of information technology via the Internet and the use of potentially sensitive patient data, an authorization was obtained, according to French law, from « Commission Nationale de l’Informatique et des Libertés » [24] and from the « Comité consultatif sur le traitement de l’information en matière de recherche dans le domaine de la santé » [25], The project was approved by the ethics committee on June the 19th, 2014. (ID RCB: 2014-A01031–46).

## Results

### Pilot study

An SMS Track pilot study was conducted to test its applicability in France. Seventeen patients were selected according to the previously described criteria over a period of 1 month. All subjects answered correctly to the SMS questions and we assumed that the study procedure would be acceptable.

### Study

#### Recruitment and compliance of clinicians

Among eleven physicians who were asked to participate, nine initially accepted to be involved (four surgeons, one medical practitioner a physiotherapist/chiropractor and three rheumatologists). Each practitioner was asked to include 50 patients during the year of inclusion, which would result in 450 patients, a sufficient number even if there would be some dropouts. Finally, only five of them included patients. One of the five stopped because of health problems before the end the of inclusion phase. Thus, three orthopaedic surgeons and one physiotherapist/chiropractor (the research officer) completed the study.

#### Recruitment of patients and compliance

Table 1 provides an overview of data in the feasibility study for shoulder pain, showing the differences between what was planned, what was obtained at baseline, and what remained at the end of the study.

**Table 1:** Overview of data in the feasibility study for shoulder pain, showing the differences between what was planned, what was obtained at baseline and what remained at the end of the study

Item	Planned	At the beginning of the study	At the end of the study
**Number of data collecting clinicians**	11 invited	9 started	4
**Sample size (patients)**	410	240 (223 + 17)	222
**SMS** **Total number of participants at beginning**	410 subjects expected to provide fortnightly responses over one year 410*26 = 10,660 SMS	223 subjects expected to provide 223 × 26 messages= 5798 SMS messages	222 subjects provided a varying number of SMS messages 33 of them not related to clinical files
**Total number of subjects with > 80% responses on at least one of the outcome variables over one year.**			190 4940 SMS
**Patient files** **at base-line complete with diagnosis**	410	206	189 (206–17)
**At follow-up at least an answer to question 15 at last follow-up**		NA	179
**Number of patients with both final response on question 15 in patient file and > 80% responses over one year on SMS**	410	NA	160 For all three questions 162 subjects for question 1 158 subjects for question 2 158 subjects for question 3

The recruitments started gradually in September 2015. The aim was to include the 410 patients over a period of 1 year, i.e. until September 2016, to have received the follow-up on the last patients by September 2017. Because the insufficient number of patients, we had to increase the inclusion period for 6 months and prolong the follow up period until March 2019. Despite this modification, the objective of 410 subjects could not be achieved. The main cause was the lack of compliance from the clinicians.

#### Data collection

Regarding the clinical files, 206 patients were included; among them 17 decided to leave the study. Finally, 189 patients were recorded with a clinical file. Of these, 109 had a complete file (initial assessment, three, six and twelve-months follow up), and 179 had information from at least the first and the last visits, with at least (the important) question 15 from the last visit, whether recorded at the medical visit or by phone.

Regarding to the SMS data, 223 started the data collection, of which one dropped out. Out of these 222 SMS files, 30 were found not to have a corresponding clinical file. Unfortunately, the SMS files included only the phone number and no information about the clinician in charge of the patient. Since phone numbers for anonymity reasons had been removed before any analysis of the files, it was impossible to track efficiently these missing clinical files. Out of the 222 files, 190 (85%) had completed at least 80% of the fortnightly text messages.

Finally, for 160 study subjects there were both a clinical file and at least 80% of the fortnightly text messages. This corresponds to 160/410 (39%) of the intended study sample, 160/222 (72%) of those who had provided any type of data at the end, and 190/222 (85%) of the subjects with SMS data.

#### Quality of data and planned analyses using either SMS data or file data or both combined

Concerning the diversity of health professionals our objective was to obtain a range of patients with supraspinatus lesions, because, it was likely that the mildest cases would preferentially have gone to a private practice while the most important cases would have gone to larger clinics and the hospital. The different geographical locations of the centers and practices were likely to contribute to the diversity of tendon lesions too.

The sample size was much lower than expected but the quality of the clinical files for the initial assessment was noted to be acceptable during data entry, but the 12 months follow-up had clearly been difficult to obtain. Nevertheless, the most important question (Question 15), on shoulder incapacity, which would be used to compare the information collected during 1 year by SMS data, was secured either by telephone interview or by mail, if the last follow-up visit data were missing.

We concluded that it would be possible to establish the pain trajectories using the SMS data over 1 year in 190 study subjects, 50 surgically treated subjects and 140 who were treated conservatively. The difference in numbers was explained by the fact that we failed to recruit a sufficient number of surgeons into the study.

#### Planned analyses using either SMS data or file data or both combined

Because of the small study sample, bi-variate analyses would be possible only for a limited number of predictor variables vs. a limited number of pain trajectory types. Nevertheless, it would be possible to visualize the SMS trajectories for numbers of days with pain, number of nights waking up due to pain, and the severity of pain over 1 year and to do this for the two treatment types (conservative vs. surgical). For the same reason (small sample size), only preliminary analyses should be performed to test the associations between baseline variables and trajectories, and to make a comparison between question 15 and SMS trajectories.

## Discussion

In this study, we collected frequent text-message data over 1 year on patients who were either treated by rehabilitation or surgical cuff repair for degeneration of the supraspinatus tendon. Although this method of data collection has been used previously with success for other musculoskeletal problems, it was the first time it was used in France and on other problems than the spine. We were interested to see, how easily it could be implemented in a culture often considered to be somewhat “non-compliant”.

### Some potential limitations should be considered

The weaknesses of this study were mainly related to the data collecting clinicians, who were non-compliant both in relation to recruiting patients and to complete correctly and return data files. This is a common phenomenon among clinicians, who are not directly implied in a research setting. This difficulty was probably worsened because the main investigator of the study was unable to accompany the clinicians appropriately over a long period of time for health reasons.

The lack of participation by clinicians has probably not biased the results in relation to severity of problem, as they had nothing to “prove” by including or excluding patients in the study. It was probably more a question of clinicians’ lack of time and interest. Clearly, all patients were not called in for the pre-planned follow-up visits or did not wish to attend. This became more marked towards the end of the follow-up period. Patients consulting an orthopaedic surgeon but not operated would probably not be interested in returning for control visits several times for a year. Fortunately, we had anticipated this difficulty by making it possible to collect the most important information through a telephone interview.

### This study has several strengths

As soon as the patient has accepted to participate, SMS data collection worked well; better than the clinical follow up. The data collection manager did not encounter any particular problems when checking that patients used the SMS system properly. In general, there were only some technical problems related to the phone or problems understanding the three questions asked. The only difficulty encountered concerned the duration of the follow-up period. Approximately 20% of the subjects in the SMS file had to be reminded and re-motivated to complete the study till the very end. Thus, their compliance was quite acceptable in relation to the text messaging, indicating that the general public find it easy to respond to text messages and that this type of study can be carried out also for shoulder problems.

The missed follow-up visits of patients either early or later related rather to the clinical files, so whatever the reason, recovery or non-recovery, this would not affect the trajectory analyses, which would be based on the SMS data that continued to be provided by the study subjects.

Other strengths of this study were that it made it possible to observe the clinical reality, as it was carried out as a multi-center practice-based project, involving ‘ordinary’ patients and clinicians, who were aided in their recruitment procedure by a clinically relevant recruitment manual. The SMS track system limits as much as possible the memory decay and also memory recall bias since the intervals between the interrogations are very short and unrelated to contact with the treating clinician. Tracking continuously the evolution of pain should provide information regarding the different patterns of pain course over time, which cannot be obtained through ordinary retrospective questionnaires.

### Recommendations

To collect information via SMS system is much easier than via clinical records. In some studies, focusing mainly on pain and function evolution, the SMS Track method deserves to be used to increase the quantity of information on large samples of patients. In addition, this method would diminish the number of clinical assessment consultations that most of the time are time consuming and the “fragile” parts of any clinical study.

## Conclusion

Our results show that French patients accepted well to provide fortnightly SMS data over 1 year and that this method of data collection was more successful than the follow-up procedures managed by their clinician, and that it could be a cheap, easy and useful alternative to track patients without the involvement of clinicians. Therefore, it should be considered for use in chronic or long-lasting disorders.

## Supplementary information

**Additional file 1.** Assessment of potential participants.

**Additional file 2.** Determination of the sample size.

## Data Availability

Data are not available for sharing as the study has not been concluded. .
