# Correction to: Feasibility study: one year fortnightly follow-up of the evolution of supra-spinatus degeneration via text-messages

**DOI:** 10.1186/s12998-020-00354-1

**Published:** 2020-11-19

**Authors:** Karl Vincent, Olivier Gagey, Charlotte Leboeuf-Yde

**Affiliations:** 1 ED 566, Université Paris-Saclay, 15 r Georges Clemenceau, 91405 Paris, Orsay, France; 2 Institut Franco-Européen de Chiropraxie, 24 Bd Paul-Vaillant-Couturier, 94200, Toulouse, Ivry-sur-Seine, France; 3 Institute for Regional Health Research, B.Winsløws, Vej 19, Dk, 5000 Odense C, Denmark

**Correction to: Chiropr Man Therap 28, 59 (2020)**

**https://doi.org/10.1186/s12998-020-00343-4**

Following publication of the original article [1], we were notified that the academic title has been mistakenly included as part of the second author’s name.

The original article has been corrected.
