# Supplementary material for: Feasibility study: one year fortnightly follow-up of the evolution of supra-spinatus degeneration via text-messages
Source: Chiropr Man Therap. 2020 Nov 4;28:59. doi: 10.1186/s12998-020-00343-4 (PMC7640667; doi:10.1186/s12998-020-00343-4)
Supplement: Supplementary file 1 — Additional file 1. Assessment of potential participants. [file 12998_2020_343_MOESM1_ESM.docx]

Additional file1: Assessment of potential participants

**The one-year evolution of shoulder pain in patients under current treatments for degeneration of the supraspinatus tendon evaluated through fortnightly text-messages**

**Practitioner code** |__|__|

**First name of the patient:** |__| (first letter) **Family name of the patient :**|__| (first letter)

| **CHIEF Investigator** | Professor  Olivier GAGEY,  PU-PH CHU de Bicêtre  Service d’orthopédie traumatologie  Université Paris-Sud  [olivier.gagey@bct.aphp.fr](mailto:olivier.gagey@bct.aphp.fr)  +336 8110 6386 |
| --- | --- |
| **Promotor** | Assistance Publique Hôpitaux de Paris  3 Avenue Victoria, 75003 Paris  Institut Franco-Européen de Chiropraxie IFEC  24 Boulevard Paul Vaillant Couturier, 94200 Ivry-sur-Seine  01 45 15 89 10 |
| **methodologist** | Professor  Charlotte Leboeuf-Yde, DC, MPH, PhD  Research Department  Spinecenter of Southern Denmark  Hospital Lillebælt  Østre Hougvej 55  DK-5500 Middelfart  Denmark |

**Practitioners’ codes**

**01 : Dr GALEA**

**02 : Dr LEININGER**

**03 : DR CASALONGA**

**04 : MR VINCENT**

**05 : DR PAVY**

**06 : DR CHAHBENDERIAN**

**07 : PR GAGEY**

**08 : Dr Lasoudris**

**09 : Dr SERROR**

| **Personal data** |
| --- |

Practitioner code |__|__| Patient initial *First name / Family name:*  |__|/|__|

Sex 🞏 M   🞏 F

Comorbidity: 🞏 Other chronic tendinous lesions, 🞏 advanced osteoarthritis, 🞏 diabetes,

🞏 Rheumatoid arthritis or other inflammatory arthritis

Treatment: 🞏 corticoid treatment over the long term

Other:

Smoker:  🞏 Yes 🞏 No

(> 10 cigarettes per day over a period of ten years minimum)

Concerned shoulder: 🞏 right, 🞏 left

Dominant shoulder  🞏 right, 🞏 left,   🞏 ambidextrous

Education level:

🞏 Level of compulsory schooling (16 years)

🞏 Bachelor's degree or above

Profession:

Professional activity: 🞏 private, 🞏 salaried

Professional activity soliciting the arm: 🞏 Yes 🞏 No

Repetitive and monotonous work: 🞏 Yes 🞏 No

Sport activity soliciting the arm:  🞏 Yes 🞏 No

Work accident concerning this shoulder: 🞏 Yes 🞏 No

Litigation concerning this shoulder: 🞏 Yes 🞏 No

| **Initial examination (1)** |
| --- |

Practitioner code |__|__| Patient initial *First name / Family name:*  |__|/|__|

**Consultation date:** |__|__|/|__|__|/20|__|__|

**History:**

Traumatic cause: 🞏 Yes 🞏 No

(Note: ordinary fall on the shoulder, uplift effort is not taken into account as trauma)

Level of pain: VAS = / 10

Constant pain: 🞏 Yes 🞏 No

Date of the beginning of the actual shoulder pain: |__|__|/|__|__|/|__|__|__|__|

Previous shoulder pain on the involved side:  🞏 Yes 🞏 No

Previous shoulder pain on the uninvolved side: 🞏 Yes 🞏 No

**Clinical examination:**

*Active arm elevation angle:* |__||__||__|*°, Painful:*🞏 Yes 🞏 No

*Passive arm elevation angle:* |__||__||__|*°, Painful:*🞏 Yes 🞏 No

**Orthopedic tests:**

Acromioclavicular pathology: 🞏 Yes 🞏 No

*(if yes: exclusion)*

Reflex sympathetic dystrophy syndrome (RSD) : 🞏 Yes 🞏 No

*(if yes: exclusion)*

Retractile capsulitis: 🞏 Yes 🞏 No

*(if yes: exclusion)*

| **Initial examination (2)** |
| --- |

Practitioner code |__|__| Patient initial *First name / Family name:*  |__|/|__|

**Complementary examination:**

***X ray*** Examination date: |__|__|/|__|__|/20|__|__|

Calcific tendinitis (> 5 mm)  Yes  No

*(if yes: exclusion)*

Glenohumeral osteoarthritis  Yes  No

*(if yes: exclusion)*

Arcromioclavicular arthropathy  Yes  No

*(if yes: exclusion)*

***Ultrasound*** Examination date :|__|__|/|__|__|/20|__|__|

**Or RMI**

Shoulder joint effusion in the bicipital groove  Yes  No

Subdeltoid bursitis  Yes  No

Isolated supraspinatus tendinopathy  Yes  No

Isolated supraspinatus entesopathy  Yes  No

Calcific tendinitis (> 5 mm)  Yes  No

*(if yes: exclusion)*

Partial supraspinatus tendon tear (superficial or articular side)  Yes  No

Complete supraspinatus tendon tear  Yes  No

Subscapular tendon lesion  Yes  No

*(if yes: exclusion)*

Infraspinatus tendon lesion  Yes  No

*(if yes: exclusion)*

 other : ……………………………………….

| **Initial examination (3)** |
| --- |

**Other complementary examination :**

**Type of the examination**: Examination date :|__|__|/|__|__|/20|__|__|

Result:

**Final diagnosis (Check only one box):**

 **Impingement syndrome**

 **Supraspinatous tendinopathy**

 **Partial supraspinatous tendon tear**

 **Complete supraspinatous tendon tear**

Final decision**:**

|  **INCLUSION** |  **EXCLUSION**  **Reason(s) for exclusion**  Presence of other pathology  No smartphone or do not know how to use it  Doesn’t want to participate to the study |
| --- | --- |

**IF INCLUSION:** Smartphone number: |__|__|__|__|__|__|__|__|__|__|

**Previous treatment(s)** (multiple choice)

 Antalgic drug

 Anti-inflammatory drug

 Injection

 Self-rehabilitation

 Rehabilitation with physical therapist

 Surgery with rehabilitation

 Arthroscopic surgery with rehabilitation

 Acupuncture

 Manual therapy

 Other : ………………………………………………………………

**Prescribed treatment**(multiple choice)

 Antalgic drug

 Anti-inflammatory drug

 Injection

 Self-rehabilitation

 Rehabilitation with physical therapist

 Surgery with rehabilitation

 Arthroscopic surgery with rehabilitation

 Acupuncture

 Manual therapy

 Other : ………………………………………………………………

**Practitioner name and signature:**

| **Mini-DASH questionary at the beginning of the follow-up** |
| --- |

Practitioner code |__|__| Patient´s initial *First name / Family name:*  |__|/|__|

Date:|__|__|/|__|__|/20|__|__|

**Instructions**

This questionnaire asks about your symptoms as well as your ability to perform certain activities. Please answer every question, based on your condition in the last week, by circling the appropriate number. If you did not have the opportunity to perform an activity in the past week, please make your best estimate of which response would be the most accurate. It doesn’t matter which hand or arm you use to perform the activity; please answer based on your ability regardless of how you perform the task

|  | **No**  **difficulty** | **Mild difficulty** | **Moderate**  **difficulty** | **Sever difficulty** | **Unable** |
| --- | --- | --- | --- | --- | --- |
| Please rate your ability to do the following activities in the last week by circling the number below the appropriate response. | | | | | |
| 1. Open a tight or new jar. | 1 | 2 | 3 | 4 | 5 |
| 2. Do heavy household chores (e.g., wash walls, floors). | 1 | 2 | 3 | 4 | 5 |
| 3. Carry a shopping bag or briefcase. | 1 | 2 | 3 | 4 | 5 |
| 4. Wash your back. | 1 | 2 | 3 | 4 | 5 |
| 5. Use a knife to cut food. | 1 | 2 | 3 | 4 | 5 |
| 6. Recreational activities in which you take some force or impact through your arm, shoulder or hand (e.g., golf, hammering, tennis, etc.) | 1 | 2 | 3 | 4 | 5 |
| 7. During the past week, to what extent has your arm, shoulder or hand problem interfered with your normal social activities with family, friends, neighbours or groups? | 1 | 2 | 3 | 4 | 5 |
| 8. During the past week, were you limited in your work or other regular daily activities as a result of your arm, shoulder or hand problem? | 1 | 2 | 3 | 4 | 5 |
|  | | | | | |
| Please rate the severity of the following symptoms in the last week. (circle number) | | | | | |
|  | **None** | **Mild** | **Moderate** | **Severe** | **Extreme** |
| 9. Arm, shoulder or hand pain. | 1 | 2 | 3 | 4 | 5 |
| 10. Tingling (pins and needles) in your arm, shoulder or hand. | 1 | 2 | 3 | 4 | 5 |
|  | **No**  **difficulty** | **Mild difficulty** | **Moderate**  **difficulty** | **Severe difficulty** | **So much difficulty that I can’t sleep** |
| **11.** During the past week, how much difficulty have you had sleeping because of the pain in your arm, shoulder or hand? (circle number) | 1 | 2 | 3 | 4 | 5 |

| Follow-up records of the responsible practitioner at 4 months (1) |
| --- |

Practitioner code |__|__| Patient initial *First name / Family name:*  |__|/|__|

**Date of consultation:** |__|__|/|__|__|/20|__|__|

**Did the patient follow the treatment?  Yes  No**

What is the actual treatment (several answers possible)?

 Antalgic drug

 Anti-inflammatory drug

 Injection

 Self-rehabilitation

 Rehabilitation with physical therapist

 Surgery with rehabilitation

 Arthroscopic surgery with rehabilitation

 Acupuncture

 Manual therapy

 Other : ………………………………………………………………

***Level of pain today***

 Severe

 Moderate

 Mild

 None

**Level of night pain during last month (frequency of night awakening)**

 No night awakening

 Some nights awakening

 Frequent nights awakening

 Every nights awakening

| Follow-up records of the responsible practitioner at 4 months (2) |
| --- |

Practitioner code |__|__| Patient initial *First name / Family name:*  |__|/|__|

***How many days did the shoulder bother you in your daily life activity during the last month?***

 0 day

 Between 1 to 7 days

 Between 8 to 14 days

 Between 15 to 21 days

 Almost every days during this period

***Evolution of shoulder pain since the last consultation***

 Important improvement

 Incomplete improvement

 No change

 Moderate aggravation

 Severe aggravation

| Follow-up records of the responsible practitioner at 6 months (1) |
| --- |

Practitioner code |__|__| Patient initial *First name / Family name:*  |__|/|__|

**Date of consultation:** |__|__|/|__|__|/20|__|__|

**Did the patient follow the treatment?  Yes  No**

What is the actual treatment (several answers possible)?

 Antalgic drug

 Anti-inflammatory drug

 Injection

 Self-rehabilitation

 Rehabilitation with physical therapist

 Surgery with rehabilitation

 Arthroscopic surgery with rehabilitation

 Acupuncture

 Manual therapy

 Other : ………………………………………………………………

***Level of pain today***

 Severe

 Moderate

 Mild

 None

**Level of night pain during last month (frequency of night awakening)**

 No night awakening

 Some nights awakening

 Frequent nights awakening

 Every nights awakening

| Follow-up records of the responsible practitioner at 6 months (2) |
| --- |

Practitioner code |__|__| Patient initial *First name / Family name:*  |__|/|__|

***How many days did the shoulder bother you in your daily life activity during the last month?***

 0 day

 Between 1 to 7 days

 Between 8 to 14 days

 Between 15 to 21 days

 Almost every days during this period

***Evolution of shoulder pain since the last consultation***

 Important improvement

 Incomplete improvement

 No change

 Moderate aggravation

 Severe aggravation

| Follow-up records of the responsible practitioner at 12 months (1) |
| --- |

Practitioner code |__|__| Patient initial *First name / Family name:*  |__|/|__|

**Date of consultation:** |__|__|/|__|__|/20|__|__|

**Did the patient follow the treatment?  Yes  No**

What is the actual treatment (several answers possible)?

 Antalgic drug

 Anti-inflammatory drug

 Injection

 Self-rehabilitation

 Rehabilitation with physical therapist

 Surgery with rehabilitation

 Arthroscopic surgery with rehabilitation

 Acupuncture

 Manual therapy

 Other : ………………………………………………………………

***Level of pain today***

 Severe

 Moderate

 Mild

 None

**Level of night pain during last month (frequency of night awakening)**

 No night awakening

 Some nights awakening

 Frequent nights awakening

 Every nights awakening

 Every nights awakening

| Follow-up records of the responsible practitioner at 12 months (2) |
| --- |

Practitioner code |__|__| Patient initial *First name / Family name:*  |__|/|__|

***How many days did the shoulder bother you in your daily life activity during the last month?***

 0 day

 Between 1 et 7 days

 Between 8 et 14 days

 Between 15 et 21 days

 Almost every days during this period

***Evolution of shoulder pain since the last consultation***

 Important improvement

 Incomplete improvement

 No change

 Moderate aggravation

 Severe aggravation

| **MiniDASH questionary at the end of the follow-up** |
| --- |

Practitioner code |__|__| Patient initial *First name / Family name:*  |__|/|__|

Date:|__|__|/|__|__|/20|__|__|

**Instructions**

This questionnaire asks about your symptoms as well as your ability to perform certain activities. Please answer every question, based on your condition in the last week, by circling the appropriate number. If you did not have the opportunity to perform an activity in the past week, please make your best estimate of which response would be the most accurate. It doesn’t matter which hand or arm you use to perform the activity; please answer based on your ability regardless of how you perform the task

|  | **No**  **difficulty** | **Mild difficulty** | **Moderate**  **difficulty** | **Sever difficulty** | **Unable** |
| --- | --- | --- | --- | --- | --- |
| Please rate your ability to do the following activities in the last week by circling the number below the appropriate response. | | | | | |
| 1. Open a tight or new jar. | 1 | 2 | 3 | 4 | 5 |
| 2. Do heavy household chores (e.g., wash walls, floors). | 1 | 2 | 3 | 4 | 5 |
| 3. Carry a shopping bag or briefcase. | 1 | 2 | 3 | 4 | 5 |
| 4. Wash your back. | 1 | 2 | 3 | 4 | 5 |
| 5. Use a knife to cut food. | 1 | 2 | 3 | 4 | 5 |
| 6. Recreational activities in which you take some force or impact through your arm, shoulder or hand (e.g., golf, hammering, tennis, etc.) | 1 | 2 | 3 | 4 | 5 |
| 7. During the past week, to what extent has your arm, shoulder or hand problem interfered with your normal social activities with family, friends, neighbours or groups? | 1 | 2 | 3 | 4 | 5 |
| 8. During the past week, were you limited in your work or other regular daily activities as a result of your arm, shoulder or hand problem? | 1 | 2 | 3 | 4 | 5 |
|  | | | | | |
| Please rate the severity of the following symptoms in the last week. (circle number) | | | | | |
|  | **None** | **Mild** | **Moderate** | **Severe** | **Extreme** |
| 9. Arm, shoulder or hand pain. | 1 | 2 | 3 | 4 | 5 |
| 10. Tingling (pins and needles) in your arm, shoulder or hand. | 1 | 2 | 3 | 4 | 5 |
|  | **No**  **difficulty** | **Mild difficulty** | **Moderate**  **difficulty** | **Severe difficulty** | **So much difficulty that I can’t sleep** |
| **11.** During the past week, how much difficulty have you had sleeping because of the pain in your arm, shoulder or hand? (circle number) | 1 | 2 | 3 | 4 | 5 |

**Additional questions to the follow-up questionaire at the end of the monitoring year (self-report):**

12. Have you had to continue performing painful movements for professional, sports or other reasons?

 Yes  No

13. How many days were you in sick leave because of your shoulder during the previous year?

|__|__|__| days

14. What treatment (s) for your shoulder have you followed for a year?

(Multiple choice)

 Antalgic drug

 Anti-inflammatory drug

 Injection

 Self-rehabilitation

 Rehabilitation with physical therapist

 Surgery with rehabilitation

 Arthroscopic surgery with rehabilitation

 Acupuncture

 Manual therapy

 Other : ………………………………………………………………

15. In the last month, what is the situation you feel describes best your difficulties in performing your activities of daily living because of your shoulder?

(**ONE** response only)

 I am constantly embarrassed (or almost)

 I have alternating periods of discomfort and calm but periods of discomfort are the most frequent

 I have alternating periods of discomfort and calm but periods of calm are the most frequent

 I do not have any problems (or almost)

| End of study sheet (responsible practitioner) |
| --- |

Practitioner code |__|__| Patient initial *First name / Family name:*  |__|/|__|

**Follow-up till the end?**  Yes  No

***If yes****,* date and sign this sheet

***If no****, specify:*

Release date of study: |__|__| /|__|__| /20|__|__|

Reason for leaving study:

 Occurrence of an exclusion criteria

 Loss of view

 Other, specify :

I,………………………………………………….the undersigned, certify that the data transcribed in the assessment sheet is conform to the medical file of the subject.

Date : |__|__| /|__|__| /20 |__|__|
