# Supplementary material for: Feasibility study: one year fortnightly follow-up of the evolution of supra-spinatus degeneration via text-messages
Source: Chiropr Man Therap. 2020 Nov 4;28:59. doi: 10.1186/s12998-020-00343-4 (PMC7640667; doi:10.1186/s12998-020-00343-4)
Supplement: Supplementary file 2 — Additional file 2. Determination of the sample size. [file 12998_2020_343_MOESM2_ESM.docx]

**Additional file 2. Determination of the sample size**

| **Sample size** |
| --- |

In the perspective of a multivariate analysis, the basic sample is estimated at 50 patients. Each possible response to the different variables studied increases the size of 10 patients (Tabachnick and Fidell, 2013)

| **Studied variables** | **Possible responses to the variables** | **Nomber of supplementary patients** |
| --- | --- | --- |
| Sex | 1. Male 2. Female | 20 |
| Age | Continuous variable | 10 |
| Sector of professionnal activity | 1. Salaried 2. Independant | 20 |
| Professional activity soliciting the arm | 1. Yes 2. No | 20 |
| Sports activity soliciting the arm | Yes  No | 20 |
| Level of education | Level of compulsory Schooling (16 years)  Bachelor's degree or above | 20 |
| Consultation sector | 1. Primary 2. Secondary | 20 |
| Type of diagnosis | 1. Impingement 2. Tendinopathy 3. Partiel tear 4. Complete tear | 40 |
| Intensité de la douleur | 1. Continuous variable | 10 |
| Durée de la douleur | 1. > 1 mois, 2. 1 - 3 mois 3. > 3 mois | 30 |
| Minidash ecore | 1. continuous variable | 10 |
| Sick leave | 1. No 2. Yes | 20 |
| Treatment | 1. Conservator 2. Surgery | 20 |
| Previous shoulder pain on the involved shoulder | 1. Yes 2. No | 20 |
| Previous shoulder pain on the uninvolved shoulder | Yes  No | 20 |
| Comorbidity | Yes   1. No | 20 |
| Smoker | Yes   1. No | 20 |
| Total |  | 360 |

The minimum sample size is therefore estimated at 410 (50 + 360) patients.
